# Wanted Fair Play for the Voluntary Hospitals

**Published:** 1891-08-29

**Authors:** 


### The Hospital

A Weekly Journal of

Science, Medicine, Nursing, and Philanthropy.

*For Hospitals, Asylums, Infirmaries, Dispensaries, Medical Practitioners, Students, Nurses, and the Charitable Public.*

Vol. X.—No. 257.] [Aug. 29, 1891.

### Wanted: Fair Play for the Voluntary Hospitals.

The Eastern Hospital scandal should draw the attention of the lay press once more to the fact that there are hospitals and hospitals. We regret to say that the writers of some of the leading articles on the report of the Local Government Board in reference to the Eastern Hospital enquiry fail to draw the distinction between this rate-supported hospital and the great voluntary hospitals of the country. The voluntary hospitals have been on their trial for two years before the Special Committee of the House of Lords, and in the result they have come out of that inquiry without any serious proof of any kind being adduced to show that the management is inefficient; indeed, the evidence proves that, on the whole, it is excellent and satisfactory. The Eastern Hospital has had an evil reputation for many years. In 1885 it was proved on oath that, as Lord Rosebery stated, it was a place flowing with milk and honey, or rather with champagne and claret and burgundy, and things which somebody liked better than milk and honey. It was consequently found that the patients there cost three times as much as those in any other hospital. As the result of the former enquiry—of the officials paid out of the rates, the Superintendent resigned; the Clerk of the Committee absconded; the Medical Superintendent was suspended; and the Clerk of the managers was cautioned. The Local Government Board, as the result of the enquiry just closed, state with respect to the food that margarine was supplied instead of butter; that fried haddock was continuously served for three weeks as the middle diet, although the diet table specified cod, brill, or sole; that the meat supply was not satisfactory; and that the potatoes were not so good as they should have been, although a high price was paid for them. The nursing arrangements are declared to be bad, and the whole management to be most discreditable to all concerned. Finally, it appears that a dance was held in a ward where there were twenty-three fever patients, and that the food supply generally was unsatisfactory.

In the result the Local Government Board state that it is impossible that the Medical Superintendent should be allowed to continue longer in the responsible position he holds, and have, therefore, required his resignation. The Matron's conduct is declared to be most reprehensible, and she is placed on probation for six months. In other words, the whole condition of the hospital has been found to be disgraceful, and to reflect most gravely upon the Local Committee and the Metropolitan Asylums Board, who are responsible for the state of affairs above recited. Such is the condition of the Eastern Hospital, and it presents an object-lesson for the information of the working and all other classes of the community as to what they may expect should the voluntary hospitals ever be placed upon the rates. The reason why the voluntary hospitals are efficient, and the rate-supported hospitals inefficient, is not far to seek. The voluntary hospitals are managed by gentlemen who give a great amount of time, and take a personal interest in their welfare. The rate-supported hospitals are managed by local committees, whose proceedings have proved on more than one occasion that individual members seem more anxious to secure contracts for their friends, than to devote their time and energies to securing efficient administration within the hospital walls. Of course fever hospitals are not so easily inspected by laymen, as those which do not take in cases which are "catching," in the popular sense of that word. Still, the present opportunity should not be lost to emphasise the fact that the voluntary hospital system is efficient, whereas the rate-supported system breaks down continually whenever it is put to the test of a public enquiry. To state, as one leading article does, that all these disclosures are extremely disquieting to those who subscribe to the great hospitals, as well as to those who have to enter them as patients, is not only unfair but untrue. No subscriptions are given to the rate-supported hospitals, of which the Eastern Hospital is a type, and as a matter of fact, hospitals supported by public subscription have always been free from scandals, such as those we are now considering. The moral, therefore, is that the lay press should carefully emphasise this fact, and so encourage the public to give their money with confidence to the voluntary hospitals.

A further point must not be lost sight of. The Commission of Enquiry appointed by the Local Government Board included the Local Government Board Inspector, who was really responsible for the state of affairs which the Commissioners' report has brought to light. It is contrary to all precedent to place one of the responsible parties in the position of a judge in an enquiry of this kind. That the report should be as strong as it is in these circumstances is not a little remarkable, and goes far to prove that the report is not only not as strong as it might have been, but not as strong possibly as the Commissioners were warranted in making it. Until the Local Government Board encourages its inspectors to write forcibly and truthfully and to insist upon much-needed reforms in the rate-supported hospitals, we must expect to find that these institutions will be inefficient, and will produce periodical scandals of the grave character brought to light by the recent investigations at the notorious Eastern Hospital.

There is one other point which concerns the honour of the Metropolitan Asylums Board. This Board consists partly of managers elected by the various Boards of Guardians in the metropolis and partly of ladies and gentlemen of position, whom it is fair to presume are credited with a special knowledge of hospital administration. In face of the repeated scandals at the Eastern Hospital and elsewhere in connection with the institutions under the control of the Metropolitan Asylums Board, the question arises whether the individual members, and especially the eighteen nominated members, can with any sense of propriety or self-respect continue to hold office, if the shameful mismanagement proved to exist by official Commissioners is to be allowed to continue. It is within our knowledge that the cause of this mismanagement is perfectly well known to most at least of the managers. It arises mainly from a bad system. We understand that at the present time each of the hospitals is under the control of a local committee, in whom is vested the appointment of the officers of the particular institution the Committee are supposed to administer, and so each officer, from the Superintendent downwards, feels that his place may depend upon his falling in with the abuses which a given committee, or its Chairman, or members may set themselves to perpetuate. This was abundantly brought out in the first enquiry in regard to the Eastern Hospital in 1885. In these circumstances the public must demand, either the resignation of the whole of the nominated members who are primarily responsible for the gross abuses proved to prevail, or else an alteration of system which shall vest the appointment of the chief officers at the asylums and hospitals under the charge of the Metropolitan Asylums Board, in the Board itself. In other words, the responsible officials must be made entirely independent of the local committees, who have heretofore neglected their duties, and proved themselves to be capable of sanctioning, if they did not even foster, evils of a character so heinous as to be probably greater, or at least as great, as those to be met with in the worst days of Bumbledom.

We, therefore, formally call upon the members of the Metropolitan Asylums Board to make this change in their system at once. If they fail to do this the public must expect that the rate-supported hospitals will continue to be mismanaged, and to prove, at the expense of the unfortunate patients who may be compulsorily removed to them, public institutions unworthy of the traditions of a great country in a civilised age like the present, of which we as a nation are prone to boast. We have shown that the Local Government Board is to blame, that the Metropolitan Asylums Board is to blame, and the report of the Local Government Board itself has proved that so far as the Eastern Hospital Committee is concerned, the whole of the members of the local committee ought to be compulsorily retired. The occasion is most important to the well-being of the poor of the metropolis, and it must not be allowed to pass, until the lessons of this particular enquiry have been driven home. Indeed we hope to have the co-operation of our colleagues in the lay press to the extent of exercising a watchful eye over the proceedings of the Metropolitan Asylums Board in regard to this report of the Local Government Board, which we give in another column, and the consideration of which has positively been referred by that Board to the very Committee which the report condemns root and branch. Anything more discouraging or better calculated to defeat all useful reform it passes the ingenuity of man to conceive or imagine.

Should anyone think we are too severe in the remarks that we have felt called upon to make in regard to the administration of these rate-supported hospitals we would venture to ask them to obtain a copy of the report of the enquiry into the Eastern Hospitals Scandals in 1885, and also to secure a copy of the report just issued. Surely the Metropolitan Asylums Board will have the courage to place printed copies of these reports at the disposal of the ratepayers on application for a small payment. If they fail to do this some metropolitan member must move for copies of these documents and see that they are issued in the form of a Parliamentary paper without delay.

